# Prevalence and risk factors of COVID-19 co-infection in TB patients

**DOI:** 10.5588/ijtldopen.25.0016

**Published:** 2025-04-09

**Authors:** T. Nyamsaikhan, G. Dorj, O. Erdenee, B. Jantsansengee, B. Badamnachin, G. Ochirdorj

**Affiliations:** ^1^ Mycobacterial Laboratory, National Centre for Communicable Diseases of Mongolia, Ulaanbaatar, Mongolia;; ^2^ Department of Epidemiology and Biostatistics, School of Public Health, Mongolian National University of Medical Sciences, Ulaanbaatar, Mongolia;; ^3^ Emergency Department, National Centre for Communicable Diseases of Mongolia, Ulaanbaatar, Mongolia.

**Keywords:** tuberculosis, Mongolia, SARS-CoV-2

Dear Editor,

In 2023, Mongolia had a high TB incidence rate, with 428 cases per 100,000 population, far above the global average of 127.^1^ By 12 July 2021, Mongolia had reported 139,205 COVID-19 cases and 678 deaths.^2^ A global cohort of 788 patients with TB and COVID-19 from 31 countries,^3^ reported that 77% achieved TB treatment success, whereas 11% died from TB, and 71% recovered from COVID-19. Mortality was higher in patients with COVID-19 before or during TB treatment, with significant risk factors including older age, HIV infection and the need for invasive ventilation.^3^ Another study cohort had 42 with active TB, seven had post-TB treatment sequelae, and the case fatality rate (CFR) was 12.3%, with higher mortality observed in older patients and those with comorbidities.^4^ A study in Mongolia comparing pre-COVID-19 (July 2018 to March 2020) versus COVID-19 (April 2020 to December 2021) periods found a reduction in new TB cases (5,989 to 4,823, *P* = 0.02), pulmonary TB cases (3,707 to 2,891, *P* = 0.006), and bacteriologically confirmed TB (2,708 to 2,177, *P* = 0.012). TB mortality decreased (*P* = 0.001).^5^ To better understand events in Mongolia, we examined COVID-19 co-infection in TB patients, using data from national databases on TB and COVID-19. Six COVID-19 phases were experienced with four major waves: imported cases (Jan–Nov 2020), cluster cases (mid-November 2020 to mid-March 2021), and waves peaking in mid-April 2021, mid-June 2021, September 2021 and January 2022.^6^ This study included TB cases registered from 1 January 2020 to 30 July 2021. Patient demographics included age, gender, employment status, and residence (urban or rural provinces). Clinical data covered medical history, TB type, diagnosis and treatment outcomes, and details on TB/COVID-19 co-infection. All cases were defined as per Mongolia's national COVID-19 and TB guidelines.^7,8^ Descriptive statistics were calculated for sociodemographic indicators. The CFR in co-infected patients was calculated as the number of deaths divided by the total number of confirmed co-infection cases, multiplied by 100. We identified the risk factors for co-infection using multivariate logistic regression analysis, calculating odds ratios (ORs) and 95% confidence intervals (CIs). The dependent variable was COVID-19 infection status, and independent variables included age, gender, employment, residency and treatment history. Differences were statistically significant at *P* < 0.05. The Ethics Review Committee, Mongolian National University of Medical Sciences, Ulaanbaatar, Mongolia, approved the study (No. 2022/3-01).

Among 5,685 TB cases, 71% were registered in 2020 and 29% in early 2021, with 5% co-infected with COVID-19. The average age of co-infected patients was 35.4 years. Employment was slightly higher among co-infected patients (5.7% vs. 4.7%, *P* = 0.001). Urban residents had a 6.8% co-infection rate, 2.7 times higher than rural residents (*P* = 0.001) (see Table). For TB alone group, most were male (67%), predominantly in urban areas (6.8% vs. 2.5% in rural areas), with a higher proportion of newly diagnosed cases (5.2%) compared to previously treated cases (3.3%), with common comorbidities, including diabetes and HIV. The COVID-19 alone group included both genders, with higher prevalence in older age groups (55–64 years: 4.3%, >65 years: 5.1%) and urban areas, and individuals with various comorbidities (such as diabetes and respiratory diseases). The co-infected COVID-TB group shared characteristics with both groups, affecting all age ranges, predominantly in urban areas (6.8% vs. 2.5% in rural areas), with a higher proportion of newly diagnosed TB cases (5.2%) and common comorbidities (Table). Among TB and COVID-19 co-infection cases, 5% had pulmonary TB, and 5% had extrapulmonary TB. Drug-resistant TB was found in 5.2% of cases. Nearly 5% were bacteriologically confirmed, and 5.2% (*n* = 103) were clinically diagnosed. The newly diagnosed group had a 2% higher incidence rate than the previously treated group (5.3% vs 3.3%), a 60.6% relative increase (*P* = 0.001).

**Table.** Risk factors for COVID-19 infection among TB patients.

Variables	Total cases	COVID-19 TB cases		
	(*n* = 5,685) *n*	(*n* = 285) *n* (%)	OR (95% CI)	*P*-value
Sex				
Female	2,467	140 (5.7)	1.3 (1.0–1.6)	0.045
Male	3,218	145 (4.5)		
Age group, years				
0–14	565	24 (4.2)	Reference	
15–24	986	60 (6.1)	0.6 (0.4–1.1)	0.1
25–34	1,323	76 (5.7)	0.7 (0.4–1.2)	0.2
35–44	1,022	42 (4.1)	1.0 (0.6–1.7)	0.8
45–54	834	39 (4.7)	0.9 (0.5–1.5)	0.7
55–64	603	26 (4.3)	0.9 (0.5–1.7)	0.9
>65	352	18 (5.1)	0.8 (0.6–1.5)	0.5
Residency				
Urban	3,285	225 (6.8)	2.8 (2.1–3.8)	0.001
Rural	2,400	60 (2.5)		
Employment status				
Employed	1,633	99 (6.1)	1.3 (1.0–1.6)	0.1
Unemployed	3,767	186 (4.9)		
Type of TB				
Pulmonary TB	3,635	181 (5.0)	1.0 (0.8–1.3)	0.79
Extrapulmonary TB	2,050	104 (5.1)		
Medical history				
Newly diagnosed	4,779	255 (5.2)	1.6 (1.1–2.4)	0.01
Previously treated	906	30 (3.3)		

Treatment outcomes for 5,545 TB cases, including 281 co-infections, were mainly successful. However, 4.9% of co-infected TB patients had unsuccessful outcomes (failure or loss to follow-up, *n* = 42), and six patients died. Among TB patients co-infected with COVID-19, the CFR was 2.1% (*n* = 6). Most were urban residents (83.3%, *n* = 5), female (66.7%, *n* = 4), with an average age of 55.8 ± 12.7 years. Of the deceased, 66.6% (n=4) were new pulmonary TB cases, and 83.3% had infiltrative TB on X-ray. *Mycobacterium tuberculosis* was detected in all cases (100%, *n* = 6) and treatment was completed in 33.3% (n=2), cured in 16.6% (*n* = 1), and unknown in 50% (*n* = 3). Additionally, 66.6% (*n* = 4) were not vaccinated against SARS-CoV-2. Females were 1.3 times more at risk of COVID-19 infection among TB patients (95% CI 1.0–1.6; *P* = 0.045), with no significant differences by age group. Urban residents were 2.8 times more at risk (95% CI 2.1–3.8; *P* = 0.0001), with no significant differences by employment status. Newly diagnosed TB patients had a 1.6 times higher risk of COVID-19 infection than previously treated cases (95% CI 1.1–2.4; *P* = 0.01) (Table). Our study found a 5% prevalence of TB cases co-infected with COVID-19. Literature shows varying prevalences, with a systematic review reporting co-infection in 38 countries,^9^ but only two studies provided prevalence rates: 0.06% in Western Cape Province, South Africa,^10^ and 0.02% in California, USA.^11^ This highlights regional differences due to TB prevalence, COVID-19 incidence and control measures.^9^ Patients in our cohort were relatively young, with an average age of 35.4 years, possibly due to higher exposure rates and social behaviours. Other studies, such as a cross-sectional analysis in California, reported a median age of 58.0 years among 91 co-infected individuals, with no significant gender difference, highlighting variability across populations.^11^

Our study found that urban areas had nearly three times more dual infections (*P* = 0.001). Similar disparities were reported for TB in Mongolia, where a WHO study from 2013–2015 found that pulmonary TB prevalence was three times higher than the annual notified rate,^12^ highlighting the need for expanded health services, especially for high-risk urban residents aged 25–44 years who did not seek medical help despite prolonged symptoms.^12^ Interestingly, the data presented here shows a higher risk of dual infection among female TB patients, contrasting with more males being detected with TB in Mongolia (2017–2022).^13^ Additionally, males with TB were less likely to seek medical care,^13^ and global studies showed higher mortality among co-infected males,^14^ while some individual studies reported increased TB mortality among females aged 25–54 years during COVID-19.^15^ This highlights the complex interplay of gender, health-seeking behaviour and infection risk.
